# Individually Perceived Parameters of Residential Infrastructure and Their Relationship with Cardiovascular Risk Factors

**DOI:** 10.3390/healthcare12192004

**Published:** 2024-10-07

**Authors:** Tatiana A. Mulerova, Timur F. Gaziev, Evgeny D. Bazdyrev, Elena V. Indukaeva, Olga V. Nakhratova, Daria P. Tsygankova, Galina V. Artamonova, Olga L. Barbarash

**Affiliations:** Research Institute for Complex Issues of Cardiovascular Diseases, 6 Academician Barbarash Boulevard, 650002 Kemerovo, Russia; mulerova-77@mail.ru (T.A.M.); gazitf@kemcardio.ru (T.F.G.); bazded@kemcardio.ru (E.D.B.); indev@kemcardio.ru (E.V.I.); cigadp@kemcardio.ru (D.P.T.); artamonova@kemcardio.ru (G.V.A.); barbol@kemcardio.ru (O.L.B.)

**Keywords:** cardiovascular disease risk factors, neighborhood design features, epidemiology

## Abstract

In modern medicine, studies devoted to the assessment of the parameters of residential infrastructure and the population’s attitude towards them have become quite large-scale. **Objectives:** The aim of the study was to establish associations between individually perceived parameters of residential infrastructure and the main modifiable cardiovascular risk factors (hypertension, obesity, lipid and carbohydrate metabolism disorders) in one of the subjects of the Russian Federation. **Methods**: The epidemiological study “Study of the influence of social factors on chronic non-communicable diseases” started in 2015 and ended in 2023. The sample was formed by using the stratification method based on the assignment to a medical organization. The study included 1598 respondents aged 35 to 70 years (491 rural residents). The study of infrastructure parameters was conducted based on the subjective opinions of respondents using the neighborhood environment walkability scale (NEWS) questionnaire, divided into eight scales. Logistic regression analysis was used to identify associations between infrastructure parameters and cardiovascular risk factors; the odds ratio (OR) and 95% confidence interval were evaluated. **Results**: Individually perceived infrastructure parameters of the scale B, reflecting the accessibility of infrastructure facilities, were associated with hypertension [OR = 1.33], obesity [OR = 1.40], and abdominal obesity [OR = 1.59]. Elements of the social infrastructure of the scale C, describing the streets in the residential area, increased the likelihood of developing obesity [OR = 1.42] and visceral obesity [OR = 1.43]. The characteristics of the residential area, represented by the scale D that evaluates pedestrian infrastructure, were associated with all major cardiovascular risk factors (hypertension [OR = 1.65], obesity [OR = 1.62] and abdominal obesity [OR = 1.82], and disorders of lipid [OR = 1.41] and carbohydrate metabolism [OR = 1.44]). **Conclusion**: Social factors represented by various aspects of infrastructure have become important criteria for determining cardiovascular health. Environmental conditions affect cardiovascular risk factors through behavioral patterns that shape the respondent’s lifestyle. Interventions in urban planning—increasing accessibility to infrastructure facilities for the population, developing a pedestrian-friendly urban environment, improving physical activity resources in areas, planning recreation areas, and landscaping—can become the most important concept for the prevention of cardiovascular diseases.

## 1. Introduction

The risk of developing chronic non-communicable diseases depends on the hereditary predisposition, the burden of risk factors, and the lifestyle that person develops under the influence of environmental factors [1]. The study of the prevalence and specificity of risk factors in the regions of the Russian Federation makes it possible to assess the general health of the population because knowing and understanding residential and regional specifics plays an important role in shaping behavior patterns [2]. At the same time, many studies do not focus on the influence of the environment on a person but on the individual perception of the environment in which he or she lives [3,4,5]. To have a healthy lifestyle, one should have much more than motivation or basic knowledge; the person also should live in appropriate conditions. In this regard, the influence of the environment and, in particular, the residential infrastructure on the health of the population has been of particular interest [6,7,8,9,10].

Available data indicates that the parameters of infrastructure located in the vicinity of residence (up to 400 m) determine the person’s habits and lifestyle, contributing to the spread of the main risk factors, primarily diseases of the circulatory system [1,2,3,4,5,6,7,8,9,10].

Research shows that general and region-specific social risk factors can determine the health of a person [6,7]. Authors evaluate all kinds of infrastructure elements: proximity to grocery stores, pharmacies, banks, restaurants, bus stops, workplaces, parks and recreation areas, pedestrian accessibility, crime rate, and the aesthetics of the residential area, which can have both a beneficial effect on public health and be risk factors [1,2,10].

The social environment represented by infrastructure, and environmental conditions that determine behavior collectively embody the social determinants of health. They contribute to cardiovascular health, which is determined by the major risk factors: arterial hypertension (AH), obesity and abdominal obesity, and disorders of lipid and carbohydrate metabolism [11]. The results of such studies can highlight indicators of social disadvantage in a particular region, their relationship with risk factors for diseases of the circulatory system, target groups with an unfavorable risk profile, and allow for the development of new preventive programs [1,2,12]. Interventions in urban planning such as increasing accessibility to infrastructure facilities for the population, developing a pedestrian-friendly urban environment, improving physical activity tools and resources, planning recreation areas, and landscaping contribute towards maintaining a healthy lifestyle, positively affecting cardiovascular health indicators of the population.

The aim of the study was to establish associations between individually perceived parameters of residential infrastructure and the main modifiable cardiovascular risk factors (hypertension, obesity, and lipid and carbohydrate metabolism disorders) in one of the subjects of the Russian Federation.

## 2. Materials and Methods

Within the framework of the Prospective Urban Rural Epidemiology (PURE) study, we started a new epidemiological study entitled “Study of the influence of social factors on chronic non-communicable diseases” in 2015. The study was completed by the end of 2023. The sample was formed by using the stratification method based on the assignment to a medical organization. The selection of households was carried out by generating random numbers using Microsoft Excel v. 2007 software. A total of 1598 respondents (477 men and 1121 women) aged 35 to 70 years were included, of which 491 were rural residents and 1107 were urban residents. We differentiated 3 age groups: the youngest respondents—people under 45 years old, the middle-aged respondents (45–64 years old), and the oldest respondents—65+ years old. To be included in the study, rural areas should have met the following requirements: the distance from the nearest city should not exceed 50 km, and the population should be at least 5000 people. Each respondent signed an informed consent form before enrollment in the study. In accordance with the principles of the Declaration of Helsinki, the Institutional Review Board of the Research Institute for Complex Issues of Cardiovascular Diseases (Kemerovo) approved the protocol of the study.

Throughout the study, we measured the respondents blood pressure according to the 2023 ESC/ESH clinical practice guidelines for the management of arterial hypertension (AH) [13]. AH was defined as a condition with systolic blood pressure ≥140 mmHg and/or diastolic blood pressure ≥90 mmHg in persons who had not received antihypertensive therapy at the time of examination and in persons with AH in history taking antihypertensive medication. Venous blood was collected from all respondents to study lipid parameters and determine the level of glycemia. Lipid metabolism disorder was defined as a deviation from the norm of any of the following indicators: total cholesterol >5.0 mmol/L, low-density lipoprotein cholesterol >3.0 mmol/L, high-density lipoprotein cholesterol <1.0 mmol/L in men and <1.2 mmol/L in women, triglycerides >1.7 mmol/L, or a combination thereof [14]. The group of people with impaired carbohydrate metabolism included respondents with fasting glycemia (glucose levels >6.1 and <7.0 mmol/L), impaired glucose tolerance (glucose levels ≥7.0 mmol/L), and diabetes mellitus [15]. Moreover, we conducted an anthropometric survey of respondents (measures of height and body weight). Obesity was defined as body mass index ≥30 kg/m^2^, abdominal obesity—waist circumference >94 cm in men and >80 cm in women [16].

We assessed the opinions of residents toward the quality of the environment using the NEWS (neighborhood environment walkability scale) questionnaire [17] (Table 1).

To ensure convenience and reliability, the questionnaire was divided into 8 scales.

The parameters of the scale A are divided into two groups depending on the accessibility: The first group includes parameters that can be reached on foot in 20 min or less; they are assigned a value of 0. The second group includes parameters that take more than 20 min to reach; they are assigned a value of 1. Respondents needed to choose one of four answers in the questions on the B–G scales: “strongly disagree”, “rather disagree”, “rather agree”, or “completely agree”. Taking into account the different phrasing of the questions on the B–G scales, the respondents answers were divided into two groups: positive and negative aspects of infrastructure. The positive aspects were assigned a value of 0, and the negative aspects were assigned a value of 1. Respondents were asked to assess their satisfaction with various living conditions in the questions of the H scale. The answers were as follows: “strongly dissatisfied”, “somewhat dissatisfied” (value 1 for the corresponding parameter), “uncertain” (respondents who chose this answer were not included in the statistical analysis), “somewhat satisfied”, and “completely satisfied” (value 0 for the corresponding parameter).

The physical activity of the study participants was assessed over the past 7 days using the International Physical Activity Questionnaire (IPAQ). Data on the number of days per week and the average time that respondents spent on walking in their free time were used. The weekly time spent on recreational walking was summarized and grouped into categories: “less than 150 min”—insufficient walking time for a healthy lifestyle, and “150 min or more”—sufficient walking time for a healthy lifestyle [13].

Statistical analysis was performed using the STATISTICA 10 software. A preliminary assessment of normality of distribution in data sets was carried out using the Kolmogorov–Smirnov test. In cases of normal distribution and equal variances in the compared groups, we used parametric tests, whereas nonparametric tests were used for non-normal distribution. Qualitative variables were represented as frequencies (percentages). The assessment of differences in indicators for two independent groups was carried out using Pearson’s chi-squared test. The Holm–Bonferroni method was used to counteract the problem of multiple comparisons. Multivariate analysis was used to establish associations between infrastructure parameters and cardiovascular risk factors. The participants with hypertension, obesity, abdominal obesity, carbohydrate metabolism disorders, and dyslipidemia were assigned a value of 1, while participants without it were assigned a value of 0. The analysis was carried out using a multifactorial logistic regression. The results of the regression analysis are presented by adjusted odds ratios (OR), 95% confidence interval (CI), and significance testing (Wald test). The *p*-value was <0.05.

## 3. Results

**Figure 1:**
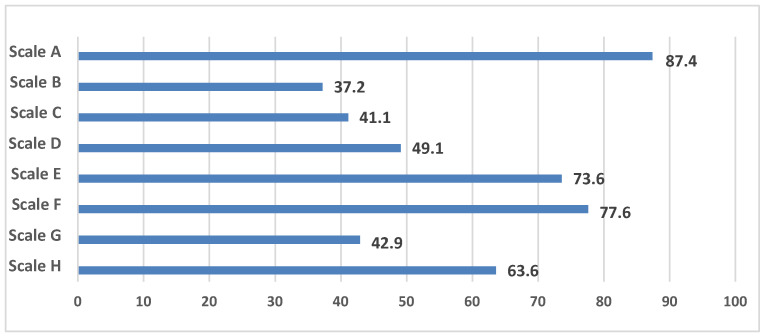
The frequency of negative perception of infrastructure parameters according to the NEWS questionnaire.

The study results revealed a highly negative attitude towards infrastructure parameters combined in the scales of the NEWS questionnaire in the presented population of adults (Figure 1). The following parameters were the most negatively perceived: from the scale A, that evaluates the walking distance between facilities, it was the remote location of work (75.3% of people); from the scale B, which assesses the accessibility of infrastructure facilities, it was a small number of facilities within walking distance (31.8%); from the scale C, which describes the streets, it was a large distance between intersections (44.4%); from the scale D, that characterizes the pedestrian infrastructure, it was the inadequate condition of the sidewalks in the residential area (41.5%); from the scale E, that evaluates the aesthetic component, it was the lack of points of interest (54.2%); from the scale F, that characterizes traffic-related safety, it was heavy traffic on the streets (59.7%); from the scale H, that describes the degree of satisfaction with certain conditions of the residential area, it was the lack of cultural and entertainment facilities (restaurants, cinemas, clubs) (41.1%).

**Figure 2:**
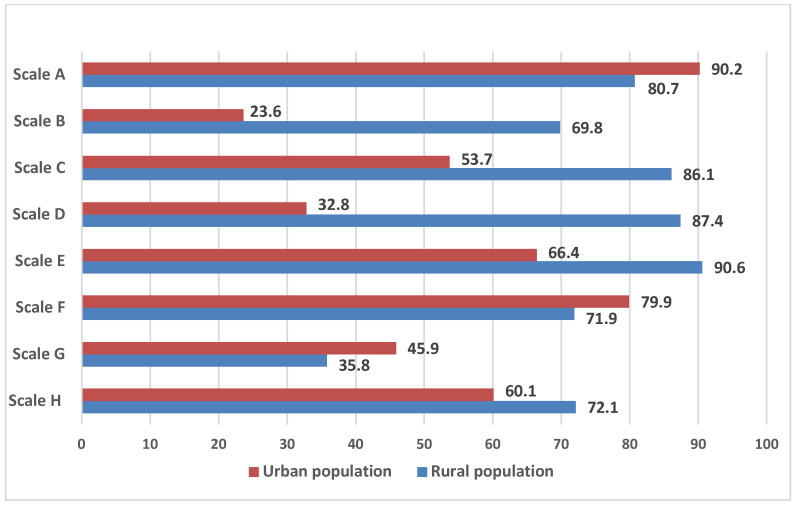
The frequency of negative perception of infrastructure parameters according to the NEWS questionnaire, depending on the place of residence.

It should be noted that the individual perception of the social parameters of the environment, combined in the scales of the NEWS questionnaire, differed among the urban and rural residents (Figure 2).

**Figure 3:**
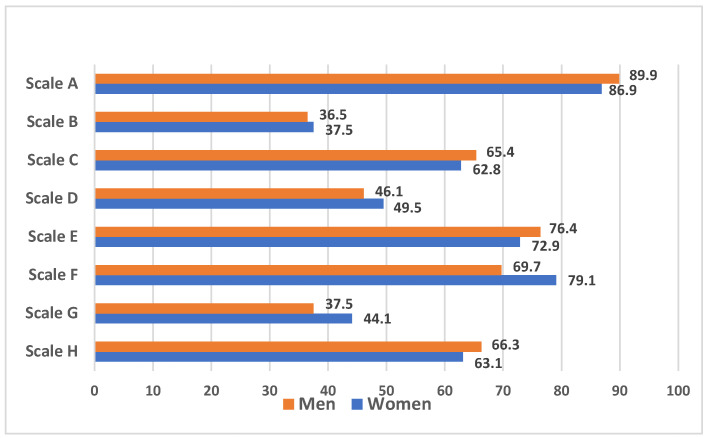
The frequency of negative perception of infrastructure parameters according to the NEWS questionnaire, depending on gender.

It should be noted that gender differences in the assessment of the parameters of residential areas were noted only for the scale F: women are more dissatisfied with busy traffic than men (79.1% vs. 69.7%; *p* = 0.002), because it makes it difficult to move along the streets (women, 61.6%; men; 50.0%; (*p* = 0.001)) (Figure 3).

Moreover, people under 45 years of age, compared with the middle-aged and older population, were more likely to show a negative attitude towards infrastructure elements of the A and G scales. In turn, middle-aged people were more likely to note dissatisfaction with the parameters from the scales D (51.2% vs. 39.5%, *p* = 0.008) and E (75.2% vs. 64.5%, *p* = 0.001) compared with the older age group (Table 2).

The prevalence of cardiovascular risk factors in the studied population of the subject of the Russian Federation (Kemerovo region), depending on gender, age, and place of residence, is shown in Table 3.

We noted the lack of physical activity as a cardiovascular risk factor in 67.5% of cases (68.1% among men, 67.3% among women (*p* = 0.779). At the same time, the incidence of this risk factor in rural areas was higher compared to the urban areas: 73.7% versus 64.8% (*p* = 0.0004), respectively. The intensity of physical activity of the population varied depending on satisfaction with the parameters of the infrastructure of the residential area (Table 4).

Risk factors such as hypertension, obesity, and abdominal obesity were more common in the rural population compared with the urban population: 75.4% vs. 66.7% (*p* = 0.001); 51.5% vs. 38.8% (*p* = 0.0001); 79.8% vs. 70.7% (*p* = 0.0001). There were no gender differences regarding hypertension: in urban residents, the incidence of AH was 69.1% in men and 65.6% in women (*p* = 0.263); in rural areas, it was 71.1% and 77.4% (*p* = 0.137), respectively. Statistically significant differences in the prevalence of AH among rural and urban residents were determined by the higher incidence among the urban population in men 65 years and older and women in the younger and middle-age groups. We noted gender differences regarding obesity: 45.7% among women, 35.9% among men (*p* = 0.0003). However, this statistical significance was achieved at the expense of respondents living in rural areas (57.5% vs. 38.3%; *p* = 0.0001), while no gender differences were found among urban residents. Abdominal obesity was more common among women than men, both in the urban population: 75.0% vs. 60.2% (*p* = 0.0001) and in the rural population: 85.9% vs. 66.4% (*p* = 0.0001). The prevalence of dyslipidemia (85.6%) did not significantly differ depending on the place of residence (urban population 85.1%, rural population 86.9%, *p* = 0.321). There were no gender differences both in the urban population (men—85.9%; women—84.7%; *p* = 0.606) and in the rural population (men—86.6%; women—87.1%; *p* = 0.875). The frequency of carbohydrate metabolism disorders was 21.8% (in the urban population 21.1%, in the rural population 23.4%, *p* = 0.292). The incidence of this pathology did not differ by gender in urban population (among men—23.2%, among women—20.1%, *p* = 0.250) and in rural population (among men—26.2%; among women—22.3%; *p* = 350).

Individually perceived infrastructure parameters of the scale B, which assesses the accessibility of infrastructure facilities, were associated with hypertension [OR = 1.33; 95% CI (1.01–1.75), *p* = 0.036], obesity [OR = 1.40; 95% CI (1.10–1.77), *p* = 0.005], and abdominal obesity [OR = 1.59; 95% CI (1.18–2.13), *p* = 0.001]. Specifically, the risk of hypertension, obesity, and abdominal obesity increased due to a long distance between public transport stops [OR = 1.77; 95% CI (1.17–2.69), *p* = 0.007], [OR = 1.71; 95% CI (1.24–2.37), *p* = 0.001], [OR = 1.89; 95% CI (1.22–2.91), *p* = 0.003]. Another parameter, the unavailability of infrastructure facilities, increased the rates of developing obesity [OR = 1.54; 95% CI (1.23–1.93), *p* = 0.001] and abdominal obesity [OR = 1.36; 95% CI (1.03–1.79), *p* = 0.029]. It should be emphasized that the likelihood of insufficient physical activity per week increased among the population that was unsatisfied with the elements of infrastructure in the scale B [OR =1.51; 95% CI (1.019–1.93), *p* = 0.001]. In this category of persons, low physical activity was detected in 40.9% of cases, and high in 31.3% of cases.

Elements of the social infrastructure of the scale C that describes streets in the residential area were associated with obesity [OR = 1.42; 95% CI (1.12–1.81), *p* = 0.003] and visceral obesity [OR = 1.43; 95% CI (1.08–1.90), *p* = 0.011]. The large distance between intersections [OR = 1.26; 95% CI (1.01–1.58), *p* = 0.039] and the lack of four-way intersections [OR = 1.42; 95% CI (1.13–1.77), *p* = 0.002] represented unfavorable infrastructure parameters and were associated with obesity. Moreover, the risk of insufficient physical activity per week increased among this population [OR = 1.27; 95% CI (1.01–1.61), *p* = 0.037].

The characteristics of the residential area, represented by the scale D evaluating pedestrian infrastructure, were associated with all major cardiovascular risk factors (AH, obesity and abdominal obesity, disorders of lipid and carbohydrate metabolism) [OR = 1.65; 95% CI (1.26–2.15), *p* = 0.0002], [OR = 1.62; 95% CI (1.28–2.04), *p* = 0.0001], [OR = 1.82; 95% CI (1.38–2.42), *p* = 0.0001], [OR = 1.41; 95% CI (1.02–1.95), *p* = 0.035], and [OR = 1.44; 95% CI (1.09–1.90), *p* = 0.009]. The lack of sidewalks in the residential area was the main element of the scale D, which determined the pedestrian inaccessibility and was associated with AH [OR = 1.66; 95% CI (1.24–2.23), *p* = 0.0001], obesity [OR = 1.75; 95% CI (1.37–2.22), *p* = 0.001] and impaired carbohydrate metabolism [OR = 1.58; 95% CI (1.18–2.12), *p* = 0.001]. It should be noted that such social elements of the scale D as lack of sidewalks [OR = 1.90; 95% CI (1.46–2.47), *p* = 0.0001], inadequate quality of sidewalks [OR = 1.55; 95% CI (1.23–1.97), *p* = 0.0002] and unsafe pedestrian crossings [OR = 1.70; 95% CI (1.23–2.34), *p* = 0.001] increased the chances of low physical activity in the population.

The parameters of the infrastructure of the scale E, characterizing the environment in the vicinity, were associated with AH [OR = 1.44; 95% CI (1.07–1.94), *p* = 0.015], namely the absence of shade from trees on the sidewalk [OR = 1.31; 95% CI (1.00–1.71), *p* = 0.048] and obesity [OR = 1.37; 95% CI (1.05–1.78), *p* = 0.016]. It should be noted that the remote location of the park increased the number of people with low physical activity (61.1% vs. 40.7%, *p* = 0.033).

In addition, the social elements of the scale H (satisfaction with certain living conditions) increased the ratio of obesity [OR = 1.33; 95%CI (1.04–1.69), *p* = 0.018]. At the same time, among the population unsatisfied with the elements of the E and H scales, the rates of low physical activity increased [OR = 1.55; 95% CI (1.20–2.01), *p* = 0.0001] and [OR = 1.37; 95% CI (1.08–1.73), *p* = 0.008].

## 4. Discussion

Studies on the assessment of the characteristics of the residential area and the individual attitudes of the population towards it have become quite extensive. According to some authors, the perceived environment of a particular area of residence affects the health of the population through the formation of behavioral habits and overall life satisfaction [5,18,19]. The results of the study by J. Liu et al. (2022) proved that a better perception of the social characteristics of the residential area contributes to an increase in physical activity, a decrease in sedentary lifestyle, smoking, and alcohol consumption [19]. Another study by TL. Gary-Webb et al. (2020) demonstrated a more favorable profile of cardiovascular disease risk factors in respondents in case of their higher satisfaction with the characteristics of the residential area [5].

Epidemiological research conducted in one of the subjects of the Russian Federation allowed us to determine the negative social indicators of the residential area associated with the main cardiovascular risk factors: remote location of work, busy traffic, lack of interesting walking areas, inaccessibility of cultural and entertainment facilities, poor condition of sidewalks. The infrastructure parameters of the scale D characterizing pedestrian accessibility were associated with all major risk factors: AH, obesity, abdominal obesity, impaired carbohydrate metabolism, and dyslipidemia. Similar results were obtained in the study by J. Zhu (2023): pedestrian accessibility determined the way the population behaves in terms of physical activity, and the lack of it contributed to the development of AH, obesity, or diabetes mellitus [20]. Several studies in recent years have revealed that the physical activity of the population is potentiated by the influence of the social environment of the residential area and is associated with obesity or abdominal obesity [21,22,23]. The environment of the residential area can play a key role in the development of the following risk factors: the absence of sidewalks in the residential area or poor-quality sidewalks lead to a decrease in daily physical activity and low physical activity in general [22]. The present study also showed low physical activity in the population unsatisfied with the pedestrian infrastructure. A similar study proved that improvements in walking areas can lead to a decrease in obesity [23]. Another study showed that the level of physical activity of residents is directly related to the characteristics of the environment—the presence of open space, the size, quality, and safety of sidewalks, the surrounding greenery, and interesting places contributed to an increase in physical activity and a decrease in weight among the population [24]. Thus, the pedestrian accessibility of the residential area is associated with a lower prevalence of cardiovascular risk factors.

It should be noted that in this study, infrastructure parameters such as the large distance between intersections and the lack of four-way intersections reduced the physical activity of the population and were associated with obesity (OR = 1.42) and visceral obesity (OR = 1.43). Data from the ELISABET database proved the association between a high pedestrian accessibility index of an area with a lower body mass index, systolic blood pressure, a lower prevalence of AH, and a higher frequency of sufficient physical activity in the population residing in the northern parts of France [25]. Similarly to the present study, research teams established associations between the risk of cardiovascular diseases calculated using biomarkers (glycosylated hemoglobin, systolic and diastolic blood pressure, high- and low-density lipoprotein cholesterol) and insufficient pedestrian accessibility in Japanese residents, primarily men [26]. In Jamaica, residents unsatisfied with pedestrian infrastructure often were obese [27]. A study conducted in the USA demonstrated a decrease in the prevalence of cardiovascular risk factors such as AH (from 35.5% to 29.7%), high cholesterol (from 34.5% to 29.2%), obesity (from 35.0% to 30.2%), and diabetes mellitus (from 11.6% to 10.6%) in cases of high pedestrian accessibility [28].

There has been a growing interest in the relationship between the accessibility of infrastructure facilities and cardiovascular risk factors [29,30]. Findings indicate that the presence of fruit/vegetable shops within walking distance was associated with low blood pressure and a low risk of circulatory system diseases, whereas the absence of shops with high-quality products within walking distance was associated with higher body weight, waist circumference, increased blood pressure, and an unfavorable cardiovascular risk profile [29,30,31]. The present study also showed an increase in the rates of developing obesity (OR = 1.54) and abdominal obesity (OR = 1.36) in the case of absence of basic infrastructure within walking distance. Similar findings were presented by R. Congdon (2019), who identified the following factors determining the development of obesity: accessibility of a healthy diet, accessibility of physical exercise, and the social environment in the residential area [31]. It should be noted that some researchers identify a link between the social disadvantage of residential areas and the risk of cardiovascular diseases through exposure to chronic stress.

## 5. Conclusions

The results of this study draw the interest of scientists to a very fascinating topic. Social factors represented by various aspects of infrastructure have become important criteria for determining cardiovascular health. Environmental conditions affect cardiovascular risk factors through behavioral patterns that shape the respondent’s lifestyle. Interventions in urban planning—increasing accessibility to infrastructure facilities for the population, developing a pedestrian-friendly urban environment, improving physical activity resources in areas, planning recreation areas, and landscaping—can become the most important concept for the prevention of cardiovascular diseases.

## Figures and Tables

**Table 1:** NEWS questionnaire.

Scales	Description
Scale A	The time spent by the respondent walking to various infrastructure facilities (pharmacies, banks, shops, etc.). Available answers: 1–5 min, 6–10 min, 11–20 min, 21–30 min, 31+ min, or I do not know.
Scale B	Accessibility of infrastructure facilities mentioned the scale A. Available answers to the questions of the B–G scales: strongly disagree, rather no, rather yes, or completely agree.
Scale C	Description of the streets in the residential area (the distance between intersections, the presence of four-way intersections, the availability of alternative routes).
Scale D	Characterization of the pedestrian infrastructure (availability and quality of sidewalks).
Scale E	Evaluation of the aesthetic component of the infrastructure (the presence of garbage, points of interest, and shade from trees).
Scale F	Characterization of traffic-related safety.
Scale G	Characterization of crime-related security.
Scale H	Description of satisfaction with certain conditions of the residential area. Available answers: strongly dissatisfied, somewhat dissatisfied, uncertain, somewhat satisfied, or completely satisfied.

**Table 2:** The frequency of negative perception of infrastructure parameters according to the NEWS questionnaire, depending on age.

Scales	Age (Years)			*p*-Value		
	<45	45–64	≥65	<45 vs. 45–64	<45 vs. ≥65	45–64 vs. ≥65
Scale A	91.7	86.2	86.4	0.020	0.057	0.951
Scale B	36.9	38.5	33.8	0.638	0.478	0.193
Scale C	64.3	64.4	57.9	0.970	0.148	0.071
Scale D	48.2	51.2	39.5	0.297	0.052	0.008
Scale E	76.5	75.2	64.5	0.687	0.003	0.001
Scale F	77.6	77.1	78.9	0.869	0.729	0.567
Scale G	49.1	42.1	39.1	0.053	0.027	0.407
Scale H	67.4	63.4	60.1	0.237	0.092	0.368

**Table 3:** Prevalence of risk factors in the population of the subject of the Russian Federation (Kemerovo region) by gender, age and place of residence.

Risk Factors	Urban Population						Rural Population					
	Male			Female			Male			Female		
	<45 Years	45–64 Years	≥65 Years	<45 Years	45–64 Years	≥65 Years	<45 Years	45–64 Years	≥65 Years	<45 Years	45–64 Years	≥65 Years
AH	56.4	74.2	77.3	32.1	68.2	90.9	44.4	72.7	95.6	49.2	81.4	93.9
O	32.7	37.9	27.3	20.7	42.1	55.1	33.3	39.4	39.1	32.8	62.8	63.3
AO	56.4	58.2	77.3	51.6	76.3	93.9	51.8	69.7	69.6	63.9	89.6	95.2
DLP	78.2	89.1	90.9	69.2	89.2	87.3	92.6	84.8	86.9	77.1	90.9	81.6
CMD	15.8	29.1	40.9	4.4	21.9	34.6	22.2	26.3	39.1	13.1	27.3	40.8

Note: AH—arterial hypertension; O—obesity; AO—abdominal obesity; DLP—dyslipidemia; CMD—carbohydrate metabolism disorders.

**Table 4:** Frequency of low/high physical activity in (%), depending on individual perception of infrastructure parameters.

Infrastructure Parameters	Low PA	High PA	*p*-Value
Remoteness of the grocery store	17.6	19.9	0.290
Remoteness of the clothing store	66.7	67.8	0.713
Remoteness of the food store	19.9	20.9	0.691
Remoteness of the bank	45.4	46.9	0.630
Remoteness of the drugstore	33.9	30.5	0.236
Remoteness of the restaurant	65.2	57.2	0.062
Remoteness of the park	61.1	40.7	0.0003
Lack of points of interests	56.9	49.8	0.013
Lack of cultural and entertainment facilities	45.3	33.9	0.0002
Remoteness of work	76.6	72.5	0.279
Remoteness of the public transport stop	13.6	14.3	0.711
Long distance between intersections	46.2	41.4	0.091
Lack of four-way intersections	45.5	39.5	0.037
Lack of sidewalks	35.6	22.5	0.001
Inadequate quality of sidewalks	45.5	34.8	0.0002
Unsafe pedestrian road crossings	19.7	12.6	0.001
Heavy traffic	56.2	54.6	0.593
Presence of litter in the vicinity	30.6	29.2	0.593
Street lighting at night	14.9	12.8	0.309
Absence of shade from trees	43.9	33.8	0.0003

Note: PA—physical activity.

## Data Availability

Data are contained within the article.
